# Transfer of the EFE-5 Executive Function Intervention Program to the Reduction of Behavioral Problems

**DOI:** 10.3390/children12050596

**Published:** 2025-05-02

**Authors:** Miriam Romero-López, Carmen Pichardo, Sylvia Sastre-Riba, Francisco Cano-García

**Affiliations:** 1 Department of Developmental and Educational Psychology, University of Granada, 18071 Granada, Spain; miriam@ugr.es (M.R.-L.); fcano@ugr.es (F.C.-G.); 2 Department of Education Sciences, University of La Rioja, 26006 Logroño, Spain; silvia.sastre@unirioja.es

**Keywords:** executive functions, behavioral problems, child education, intervention, transfer, prevention

## Abstract

Background/Objectives: Numerous research studies link the improvement in executive functions and school success. However, there is hardly any research analyzing the transfer of this improvement to behavioral problems. This study analyzed whether improving executive functions, through contextualized daily activities, decreases these behaviors. Methods: Fifty third-year kindergarten students participated, divided into experimental and active control groups, with pre- and post-intervention measurements. The students in the experimental group were trained with the EFE-P program and the students in the control group received regular curriculum activities. The EFE-P program (i) has been designed with the aim of improving their executive functions, using a game-based approach; (ii) not only involves cognitive activities, but also behavioral and emotional activities, related to the warm aspects of executive functions; and (iii) consists of three units (inhibitory control, working memory and cognitive flexibility), with each unit involving 7 sessions (21 sessions in total), with an approximate duration of 30 min each. Results: Analysis of the data using a generalized linear mixed effects model revealed that students in the experimental group scored lower for behavioral problems than those in the active control group and the effect sizes were large for all of them: aggressiveness (*d* = 1.25); hyperactivity (*d* = 0.77); attention deficit (*d* = 1.12); anxiety (*d* = 0.82); and depression (*d* = 1.51). Conclusions: After discussing the results, it is concluded that intervention in executive functions induces, by way of distant transfer, a decrease in behavioral problems in preschool; the role of contextualized activities in real situations is emphasized; and several implications for practice and research are discussed.

## 1. Introduction

There is ample empirical evidence on the key role of executive functions in school readiness, social–emotional development, and school performance [1,2]. Conversely, deficits in these functions appear to be linked to various school-related problems (e.g., behavioral, underachievement) [3]. All this has led researchers to pay special attention to the training of executive functions through intervention programs [4,5,6,7,8].

### 1.1. Executive Functions

Executive functions refer to high-level cognitive processes [9] that, as orchestrators of intellectual abilities, allow considering goals and carrying them out through their planning and monitoring, ignoring those behaviors, thoughts, emotions and feelings that interfere with their achievement [10]. Three are the key components of these processes [9,11,12]:

(i) Inhibitory control refers to people’s ability to control their thoughts, emotions, and behaviors. Inhibitory control allows us to choose how to react and behave instead of automatically following impulses, habits of thought or action, and environmental stimuli. Although it is not easy, having this ability offers the possibility to change and avoid embarrassing situations.

(ii) Working memory is related to people’s ability to keep information active in the mind and to be able to retain it without the stimulus being present. It is essential for reasoning and problem solving, as it requires having a large amount of information in mind, analyzing their relationships, and possibly reorganizing them to form new combinations. Working memory is necessary to process events that occur over time, as it always requires retaining previous information and linking it to what is happening in the present.

(iii) Cognitive flexibility allows people to generate different solutions to a problem and to be able to combine the satisfaction of their own desires with the interests of others. The ability to adapt to changes in demands or priorities, accept mistakes, and take advantage of unexpected opportunities are important aspects of cognitive flexibility [11].

### 1.2. Behavioral Problems and Executive Functions

According to Achenbach [13], behavioral problems are grouped into two categories (internalizing and externalizing). Internalizing problems are of internal origin and refer to problems that generate discomfort, tension, and suffering in the individual. They include problems such as depression, anxiety, isolation, inhibition, or somatic complaints. Externalizing problems are directed to the outside, generating discomfort in other people. They are related to defiant, disruptive, or maladaptive behavior. Both internalizing and externalizing problems can produce problems in social interaction and adaptation. In this respect, externalizing problems are related to aggressive responses and antisocial behaviors. Internalizing problems involve difficulty managing emotions and cognitions.

Behavioral problems have great personal and social significance, proving to be a risk factor for the development of multiple negative behaviors in adolescence and adulthood [14,15,16]. These include, for example, school dropout, antisocial behavior, emotional disorders, or the use of violence [17,18,19]. Risk factors for antisocial behavior are associated with personal, family, school, or contextual variables, with impulsivity and attention problems standing out among the former, generally related to deficits in executive functions [3].

The relationship between executive functions and behavioral problems has been the focus of several theoretical and empirical investigations. Some authors have argued that there is a reciprocal development of executive functions and self-regulatory capacity [20], while others have suggested that executive function provides the basis for more advanced behavioral regulation [21]. According to the latter, the ability to regulate thoughts and attention serves to help boys and girls control their behavioral responses to social stimuli [22].

In the case of preschool students, because they are in the early stages of development, they usually show difficulties in the three key components inherent to executive functions:

(i) Inhibitory control: Difficulties in inhibitory control are often observed in preschool students [23], related, for example, to avoiding inappropriate comments; not touching attractive toys in a store, despite their parents’ warning; or waiting their turn when they are playing. These difficulties seem to be related to behavioral problems related to socialization. In this sense, the results of the research by Caporaso et al. [24] suggest that preschoolers who present good inhibitory control are generally more accepted and, therefore, less rejected by their peers. On the contrary, students who present difficulties in inhibiting their impulses are more likely to exhibit behaviors such as physical or verbal aggression and may be considered as bad playmates by the rest of their peers [25]. As inhibitory control develops, preschool students learn to respond more moderately to emotional events and to control their behaviors and responses so that they are more appropriate and adaptive [26]. In addition, the ability to delay reward is important for regulating emotions, as it allows them to control their emotional and behavioral responses in situations where the desire for a reward is high (e.g., being able to wait for a desired toy without exhibiting behavioral problems) [27].

(ii) Working memory: This also seems to be related to a lower probability of behavioral problems. The reason lies in the fact that it makes it easier for schoolchildren to remember which behaviors are appropriate and socially accepted and which are inappropriate and, therefore, should be avoided; therefore, it may act as a protective factor against behavioral problems [11]. In this sense, according to some authors (e.g., Garon et al. [26]), the development of working memory throughout the preschool years facilitates students’ recall and application of strategies to regulate emotions.

(iii) Cognitive flexibility: This facilitates, from early childhood, the ability of people to use dialog instead of aggression [28]. Thus, the better social adjustment of young children with better executive functioning may be explained, in part, by their competence in the use of constructive means of conflict resolution such as, for example, inhibition of aggressive responses or cognitive flexibility to perform new, more adaptive behaviors [29]. Along these lines, Hughes and Ensor [30] found significant associations between deficits in preschool cognitive functions and early problem behaviors (e.g., absence of regulation).

Most of the research on the relationship between executive functions and behavioral problems tends to be correlational. Therefore, they share the limitation that despite focusing on analyzing the mutual relationships between variables, de facto, researchers cannot establish causal links between them. This has already been pointed out by Farrington [3] (2005) who, moreover, emphasized the importance of preventing problem behaviors through intervention programs.

### 1.3. Executive Function Intervention Programs

In recent years, numerous activities and programs have been designed to optimize executive functioning from childhood onwards. Among the most widely used programs are “Tools of the Mind” [4] and “Cogmed Working Memory Training” [31]. The main objective of the first one is to encourage students to regulate their own behavior, and includes activities that improve emotional, social, and cognitive self-control through fantasy, attention, and memory games, and speech regulation activities. The second one, on the other hand, has as its main objective the improvement of working memory through computerized training.

There are several investigations whose results have demonstrated the possibility of improving executive functions in the early childhood education stage, thanks to the implementation of programs [6,7,8,32,33]. However, a major limitation of these is that very few of them [34] have focused on analyzing whether executive improvement is transferable to other contexts, beyond the one in which the program was implemented. In addition to this, there are currently few studies that have focused on the transfer of executive improvement through school programs aimed at reducing problem behaviors, and the existing ones [6] are not aimed at the early childhood education stage.

In the meta-analysis by Kassai et al. [35] near transfer was defined as the improvement of the trained executive component (e.g., improvement of working memory when training this skill) and far transfer as the improvement of skills not directly trained (e.g., improvement of academic performance when training working memory). The results of that meta-analysis, like that of other similar ones [36,37,38], have shown that, although working memory is amenable to improvement, it is not transferable to domains such as social, academic, or emotional.

This absence of distant transfer could be due to a possible limitation of the training programs used, since they did not include activities or aspects of daily life that facilitate such transfer. That possible explanation is supported by the fact that, in the investigations in which those were included, the presence of distant transfer was detected, in the sense that the improvement of executive functions impacted positively on academic performance [5,6], peer help, and cooperation [5] and adaptation [6].

In this line, it is important to highlight one of the conclusions drawn by Diamond and Ling [39], after analyzing 84 studies in which different programs for the improvement of executive functions were applied. That conclusion was that practice leads to the improvement of these functions and their application in other contexts where they are needed. However, this improvement is only focused on the skills that are practiced, and no transfer to other skills was observed.

This important limitation has been overcome in the so-called Executive Function Training Program in Preschool (EFE-P). The EFE-P, applied in the present research, is characterized by its (i) ease of inclusion in the regular early childhood education curriculum, (ii) applicability by teachers in any socio-cultural context, (iii) transferability to social competence [40], and (iv) use of daily life activities to foster executive functions (e.g., it is an ecological program).

### 1.4. The Present Investigation

In view of the limitations detected in the research on the relationship between behavioral problems and executive functions, as well as on the training of the latter through intervention programs, it was considered appropriate to (i) focus the research on the behavioral problems of preschool students; (ii) apply an intervention program, such as the EFE-P, that included daily activities; (iii) adopt an experimental design that would allow going beyond mere correlations; and (iv) examine whether the program induced a decrease in the students’ problem behaviors.

Therefore, the aim of the present investigation was to analyze, by means of an experimental design, whether the improvement of executive functions through the EFE-P program leads to a distant transfer effect on the reduction in conduct problems. We hypothesized that preschoolers who participate in the EFE-P program will decrease their conduct problems compared to their peers who do not participate in the program.

## 2. Materials and Methods

### 2.1. Participants

The participants (N = 50) were preschoolers in the third year of kindergarten, aged 5/6 years (*M age* = 5.5 years, *SD age* = 0.23). According to the data provided by the center, all were of Caucasian/European ethnicity, the family socioeconomic level was average (about EUR 26,000 net, e.g., around the national average), and they attended a single educational center in Granada (Spain).

The prerequisites for participation were (i) no history of chronic illness and/or psychopathology and (ii) typical or corrected sensory capacity. During recruitment, 5 of the initial participants did not meet these requirements and were discarded, resulting in a final sample of 50 preschoolers.

The active control group (N = 25) consisted of 13 boys and 12 girls (*M age* = 5.50 years; *SD age* = 0.26), while the experimental group (N = 25) consisted of 12 boys and 13 girls (*M age* = 5.46 years; *SD age* = 0.20). Participants were assigned to these groups individually and randomly, although the sex variable was blocked so that both groups were made up of a similar number of boys and girls.

In this way, each boy and girl was assigned a number and was randomly assigned, according to gender, to one of the control or experimental groups.

### 2.2. Instruments

The Behavior Assessment System for Children scale (BASC) [41,42] was used to assess behavioral problems. For the research, the Teacher Rating Scale (TRS) was used. This instrument includes 14 clinical scales, of which only the following were used: aggression (13 items, e.g., “He hits other children”); hyperactivity (16 items, e.g., “He yells”); attention problems (8 items, e.g., “He forgets things”); anxiety (7 items, e.g., “He gets very upset when he loses something”); depression (11 items, e.g., “He is sad”). The items are answered according to a Likert-type scale (from 0 = never to 3 = almost always). The internal consistency indices of the instrument obtained in the present investigation are as follows: aggressiveness α = 0.93; hyperactivity α = 0.93; attention problems α = 0.90; anxiety α = 0.67; depression α = 0.85.

### 2.3. Procedure

Prior to the start of the research, the project was submitted to and approved by the Bioethics Committee on Human Research of the University of Granada (Code: 2420/CEIH/2021), after its compliance with the Data Protection Act and the Code of Ethics in Psychology.

Subsequently, all the early childhood education centers in the city of Granada (Spain) were consulted to find out if the school management of each one of them was interested in the training and application of the EFE-P program. From among all the interested centers, a random selection of an educational center was made in order to control for possible variables such as educational method, center philosophy, physical space, educational activities, and demographic characteristics.

Both the center’s management and faculty gave their written consent to conduct the research. After this, the family members and teachers involved were summoned to a meeting in which both the objectives of the investigation and the procedure to be followed were explained to them. At the end of the study, all the attending family members signed a written consent for their sons and/or daughters to participate in the study. Those who did not attend received an informative letter explaining all the points discussed at the meeting and were asked to return it signed, if they agreed. Again, all gave written consent to participate.

**Figure 1:**
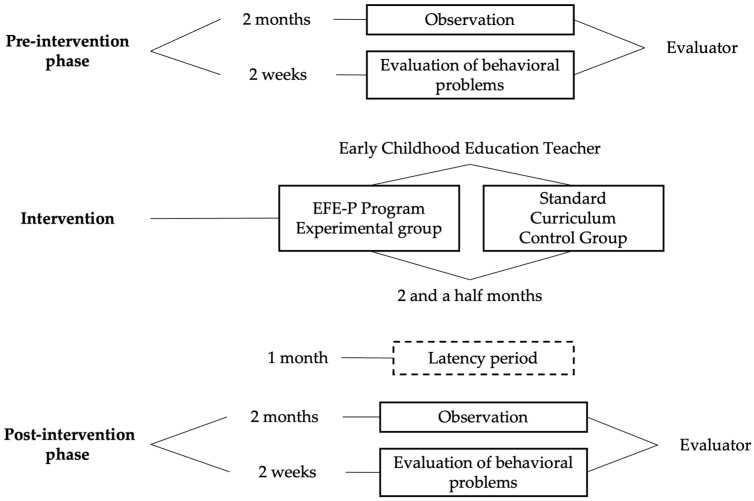
Temporal distribution of the study phases.

This study required the hiring of an evaluator and an early childhood education teacher and involved three phases (see Figure 1): pre-intervention, intervention, and post-intervention.

- Pre-intervention phase

In this stage, the evaluator received training on the different behaviors to be observed and evaluated and recorded the observed behaviors using a form (one for the classroom and one for recess) (2 months). For this purpose, he conducted four-hour sessions, on similar days and at similar times, for all participants, in order to ensure that both the students in the experimental group and the active control group were observed under similar conditions. Subsequently, according to the annotations collected, he completed the BASC scale (2 weeks) [41].

At the same time, the early childhood education teacher received training on how to properly apply the EFE-P program in the experimental group (2 months). The EFE-P program (i) has been designed for students aged 5 to 6 years old, with the aim of improving their executive functions using a game-based approach, so that participants learn while having fun; (ii) not only involves cognitive activities, but also behavioral and emotional activities, related to the warm aspects of executive functions; (iii) consists of three units (inhibitory control, working memory, and cognitive flexibility), with each unit involving 7 sessions, with increasing difficulty; and (iv) therefore includes 21 sessions, with an approximate duration of 30 min, which are carried out twice a week.

The activities included in the EFE-P are linked to the transfer of executive functions to everyday life situations and share a common basis: to pose a hypothetical situation of interaction between peers where a deficit in one of the functions that it enhances is reflected, the basis on which different types of sessions are organized. Some sessions begin with an activity in which the teacher tells a fantasy story centered on two characters, Carla and Pepe, two friends who have trouble thinking before they act. For example, Carla pushes Pepe because she wants to be first in line and Pepe hits Carla.

Subsequently, questions are asked about how they would act in a similar situation and what the characters should have done to solve the problem in a more positive way. Other sessions begin with popularly known traditional stories, modifying the roles of the characters in order to increase cognitive flexibility. For example, the teacher tells a transformed version of the three little pigs, where the good guy is the wolf and the bad guys are the three little pigs.

The EFE-P also includes plastic, motor, auditory, and visual activities. For example, (i) students are asked to draw a picture and when the teacher blows the whistle they switch with their partner on the right and continue drawing their partner’s picture, or (ii) the teacher makes the sound of different animals, and the students must repeat them in the same order.

The contract teacher was also the one who implemented the regular curriculum activities in the active control group (e.g., construction games, stories, drawings). However, to ensure an unbiased performance, a double-blind method was applied, whereby both she and the evaluator were not informed of the research objectives.

- Post-intervention phase

Here, the evaluator repeated the observation period (2 months), both in the classroom and on the playground, and then completed the BASC scale again [41].

### 2.4. Statistical Design and Analysis

In accordance with the objectives of the study, an individual randomized trial experimental design was applied, with two groups (experimental and active control) and two evaluation times (pre-intervention and post-intervention).

The analyses performed were of two types. First, descriptive analyses were carried out (basically, means and standard deviations) of the variables considered. Second, given the sample size (relatively small) and especially the design (repeated measures), a Generalized Linear Mixed Model (GLMM) analysis with logit link function and Poisson distribution with a random intercept for each participant was used to compare the two groups in pre and post measurements. The reasons for using the GLMM were numerous: (i) it allows extending the classical linear fixed effects model by including random effects for the analysis of data with possibly non-normal distributions and (ii) it makes it possible to model the structure of errors in data from longitudinal measurements [43]. Third, the effect size of the differences between the experimental and active control groups was calculated. Considering that there was no normal distribution of the data for the different variables, a nonparametric test of difference in means (Mann–Whitney U) was performed to obtain the effect size of the intervention, using the resulting data to calculate Cohen’s d. Large (*d* ≥ 0.80), medium (0.50 ≤ *d* ≤ 0.79), and small (0.20 ≤ *d* ≤ 0.49) effects are estimated [44].

All analyses were conducted using SPSS 24 (IBM Statistics, 2016, Armonk, NY, USA: IBM Corp).

## 3. Results

### 3.1. Descriptive Analysis

The results of the descriptive analyses corresponding to the variables of the participants’ behavioral problems, grouped according to the type of group and time of observation, are shown in Table 1. In both groups (intervention and active control), the mean scores obtained were lower at time 2 (post-intervention) compared to time 1 (pre-intervention).

### 3.2. GLMM Analysis

Considering that the main objective of the research was to analyze whether the improvement of executive functions through the EFE-P program leads to a distance transfer effect in the reduction in conduct problems, analyses were carried out for two groups (experimental and active control) and two evaluation moments (pre-intervention and post-intervention). The objective was to analyze whether in the post-intervention phase the students who participated in the EFE-P program obtained lower scores in behavior problems than their peers who participated in the standard curricular program. In this way, it would be demonstrated that training in executive functions produces a reduction in behavioral problems through transfer.

The results of the analysis of this model show statistically significant differences in the five behavioral problem variables, both in relation to the type of group and at the time of observation (Time), as well as in terms of the Group x Time interaction.

In this sense, the results are as follows: aggressiveness, Group (*F*(1,196) = 27.56, *p* < 0.001), Time (*F*(1,196) = 229.07, *p* < 0.001), and Group x Time (*F*(1,196) = 91.30, *p* < 0.001); hyperactivity, Group (F(1196) = 12.97, *p* < 0.001), Time (*F*(1,196) = 165.14, *p* < 0.001), and Group x Time (*F*(1,196) = 64.38, *p* < 0.001); attention problems Group (*F*(1,196) = 29.14, *p* < 0.001), Time (*F*(1,196) = 170.46, *p* < 0.001), and Group x Time (*F*(1,196) = 63.51, *p* < 0.001); anxiety, Group (*F*(1,196) = 35.27, *p* < 0.001), Time (*F*(1,196) = 268.85, *p* < 0.001), and Group x Time (*F*(1,196) = 109.59, *p* < 0.001); depression, Group (*F*(1,196) = 37.32, *p* < 0.001), Time (*F*(1,196) = 152.38, *p* < 0.001), and Group x Time (*F*(1,196) = 40.25, *p* < 0.001).

The results of the analysis of the difference in means according to the type of group (experimental and active control) and the time of observation (Time 1 and Time 2) are shown in Table 2.

The results presented in Table 2 reveal that the differences in the five behavioral problem variables studied depend on the group analyzed. Participants in the active control group showed significantly higher scores than the experimental group on these five variables. Effect sizes were large on all of them: aggressiveness (*d* = 1.25); hyperactivity (*d* = 0.77); attention deficit (*d* = 1.12); anxiety (*d* = 0.82); and depression (*d* = 1.51).

In contrast, the differences in the five behavioral problem variables do not seem to depend on the timing/phase of the intervention, as regardless of the group, participants obtain higher scores in the pre-intervention phase (Time 1) versus the post-intervention phase (Time 2).

## 4. Discussion

The aim of the present research was to analyze whether the improvement of executive functions in early childhood education, through an intervention program that included activities contextualized in real situations, could be transferred in a distant way to the reduction in behavioral problems of the participants.

Although the participants’ executive functions were not evaluated and it is not possible to verify directly whether the changes in behavioral problems are associated with the changes that the participants obtain from the intervention in executive functions, due to the intervention program, the results obtained confirm the hypothesis proposed, since all the participating students, especially and significantly those in the experimental group, reduced their behavioral problems in the post-intervention phase.

The fact that all students show signs of a decrease in these problems could be explained in part by the fact that preschoolers are in the midst of cognitive and social development. Moreover, because the objective of the school at this educational stage focuses on enhancing social development and the teaching of coexistence rules, this could have resulted in a decrease in student behavior problems [45].

However, students in the experimental group, after their participation in the intervention program, are less hostile, show less physical and verbal aggressiveness, are less hyperactive, reflect before acting, obtain lower scores in depression and anxiety, are less distracted, and are able to spend more time concentrating, compared to their peers in the active control group.

Therefore, the proven efficacy of the EFE-P program to increase executive functions [33], whose deficit has been linked in some studies [30,46] with behavioral problems in preschoolers, is transferred in a distant way, evidencing a clear decrease in these.

Our results are consistent with those obtained in other research in which intervention to optimize executive functions also improved other areas of behavior, generating a distant transfer in areas such as academic and behavioral skills [6], peer inclusion, and students’ ability to get along and be kind and helpful to each other [5]. What is interesting about these investigations is that they share with the present research the fact that the intervention programs are applied in the context of the classroom, in regular classes with regular teachers. In addition, they include activities in which executive functions play a relevant role in successfully coping with the demands of daily life in childhood contexts. In other words, the common denominator is the investigation of the efficacy of interventions in the “real world”, the very context where executive functions are to be applied and must show their usefulness.

Our results, on the contrary, differ significantly from those mentioned in various investigations included in several meta-analyses [35,36]. An analysis of these research designs reveals that, although the intervention programs included executive function training and some activities, these did not turn out to be typical of daily life. In other words, these investigations used activities that were decontextualized from the real world. For example, the night–day task, which, despite being aimed at improving inhibitory control and cognitive flexibility, is not in any way an executive task used in the day-to-day life of preschoolers and, consequently, is complicated and difficult to generalize to the real world.

The results of the present study, although certainly significant, are not without some limitations. In the first place, the participants’ executive functions were not assessed before and after the intervention, to ensure more clearly that the changes produced by the participation in the EFE-P program in behavioral problems were due to the improvement in executive functions. To verify this effect, a multivariate design including executive functions in addition to conduct problems should have been used. Secondly, its generalization to other contexts is complex and difficult, since the research was conducted in a single educational center, with preschoolers aged 5–6 years. Therefore, it would be interesting in the future to replicate the study in other contexts, as well as to extend the number of participant and the age range analyzed. Thirdly, the observation and evaluation of student behavior were carried out by a single person in each case, using a single evaluation instrument. Therefore, in future research, it would be interesting to consolidate the information collected by including inter-observer evaluation and multiple methods of evaluation, for example, family members or teachers. Finally, it would also be interesting to analyze the long-term impact of the program and to include other variables that could act as mediators between cognitive development and behavioral problems, such as, for example, the parents’ educational styles.

The findings of the present research have therefore contributed substantially to (i) expanding the existing knowledge on the role of executive function training in the reduction in school-related behavioral problems (e.g., aggression, attention deficit, depression) and (ii) emphasizing the importance of daily activities in this training and its subsequent influence on the reduction in the aforementioned problems.

From these findings, it is possible to derive some implications for both research and practice. In relation to research, it seems promising to use experimental designs for repeated data, and to model these statistically using GLMM, in order to increase the drawing of causal inferences as much as possible. In relation to practice, everything points to the need for interventions to improve executive functions to include, as a requirement, the inclusion of contextualized activities in real situations, as they significantly facilitate distant transfer.

## Figures and Tables

**Table 1:** Descriptive data of behavioral problems by groups and times.

	Moment/Phase/Time of Observation					
	Time 1 (Pre-Intervention)			Time 2 (Post-Intervention)		
Groups and Problems	*M* (*SD*)	*Min.*	*Max.*	*M (SD)*	*Min.*	*Max.*
Group 1 (Experimental)						
Aggressiveness	0.48 (0.07)	0.00	1.75	0.09 (0.02)	0.00	0.58
Hyperactivity	0.58 (0.07)	0.00	2.20	0.14 (0.03)	0.00	0.70
Attention problems	0.72 (0.06)	0.00	1.57	0.14 (0.02)	0.00	0.71
Anxiety	0.47 (0.05)	0.00	1.29	0.03 (0.01)	0.00	0.29
Depression	0.25 (0.03)	0.00	0.82	0.06 (0.01)	0.00	0.45
Group 2 (Active control)						
Aggressiveness	0.79 (0.07)	0.08	1.83	0.67 (0.06)	0.00	1.75
Hyperactivity	0.76 (0.09)	0.00	2.20	0.70 (0.08)	0.00	2.10
Attention problems	1.10 (0.09)	0.00	2.43	0.93 (0.08)	0.00	2.00
Anxiety	0.54 (0.05)	0.00	1.43	0.46 (0.04)	0.00	1.29
Depression	0.60 (0.05)	0.00	1.64	0.49 (0.04)	0.00	1.36

Note: *M* = mean; *SD* = standard deviation.

**Table 2:** Pairwise contrast of the Group–Time interaction, using the means estimated by the model on the program variables.

				IC 95%	
	*Estimate (DT)*	*t*	*p-Value.*	*L. Lower*	*L. Superior*
Aggressiveness					
Group 1–Group 2	−5.46 (0.35)	−5.28	<0.001	−7.50	−3.43
Time 1–Time 2	2.62 (0.27)	9.77	<0.001	2.09	3.15
Hyperactivity					
Group 1–Group 2	−3.20 (0.87)	−3.66	<0.001	−4.92	−1.48
Time 1–Time 2	2.15 (0.25)	8.54	<0.001	1.66	2.65
Attention problems					
Group 1–Group 2	−4.05 (0.64)	−6.29	<0.001	−5.32	−2.78
Time 1–Time 2	2.54 (0.22)	11.42	<0.001	2.10	2.98
Anxiety					
Group 1–Group 2	−2.23 (0.33)	−6.79	<0.001	−2.88	−1.59
Time 1–Time 2	1.52 (0.10)	14.45	<0.001	1.31	1.72
Depression					
Group 1–Group 2	−4.02 (0.68)	−5.94	<0.001	−5.35	−2.69
Time 1–Time 2	1.45 (0.14)	1.36	<0.001	1.18	1.73

Note: *SD* = standard deviation; *CI* = confidence intervals; *L* = limit; Group 1 = intervention; Group 2 = active control; Time 1 = pre-intervention; Time 2 = post-intervention.

## Data Availability

The data sets presented in this article are not readily available due to data protection of study participants.

## References

[1] Takacs Z.K., Kassai R. (2019). The efficacy of different interventions to foster children’s executive function skills: A series of meta-analyses. Psychol. Bull..

[2] Riccio C.A., Ca M.J., Worrell F.C., Hughes T.L., Dixson D.D. (2020). Executive function and school performance. The Cambridge Handbook of Applied School Psychology.

[3] Farrington D.P. (2005). Childhood origins of antisocial behavior. Clin. Psychol. Psychother..

[4] Bodrova E., Leong D.J. (2007). Tools of the Mind: The Vygotskian Approach to Early Childhood Education.

[5] Diamond A., Lee C., Senften P., Lam A., Abbott D. (2019). Randomized control trial of Tools of the Mind: Marked benefits to kindergarten children and their teachers. PLoS ONE.

[6] Dias N.M., Seabra A.G. (2017). Intervention for executive functions development in early elementary school children: Effects on learning and behaviour, and follow-up maintenance. Educ. Psychol..

[7] Espinet S.D., Anderson J.E., Zelazo P.D. (2013). Reflection training improves executive function in preschool-age children: Behavioral and neural effects. Dev. Cogn. Neurosci..

[8] Traverso L., Viterbori P., Usai M.C. (2015). Improving executive function in childhood: Evaluation of a training intervention for 5-year-old children. Front. Psychol..

[9] Friedman N.P., Miyake A. (2017). Unity and diversity of executive functions: Individual differences as a window on cognitive structure. Cortex.

[10] Santa-Cruz C., Rosas R. (2017). Mapping of executive functions. Estud. Psicol..

[11] Diamond A. (2013). Executive functions. Annu. Rev. Psychol..

[12] Miyake A., Friedman N.P., Emerson M.J., Witzki A.H., Howerter A., Wager T.D. (2000). The unity and diversity of executive functions and their contributions to complex “Frontal lobe” tasks: A latent variable analysis. Cogn. Psychol..

[13] Achenbach T.M. (1995). Empirically based assessment and taxonomy: Applications to clinical research. Psychol. Assess..

[14] Kassing F., Godwin J., Lochman J.E., Coie J.D. (2019). Using early childhood behavior problems to predict adult convictions. J. Abnorm. Child Psychol..

[15] Major S.O., Seabra-Santos M.J., Martin R.P. (2022). Differentiating preschoolers with (out) social-emotional and behavioral problems: Do we have a useful tool?. Assess. Eff. Interv..

[16] Yang Y., Shields G.S., Zhang Y., Wu H., Chen H., Romer A.L. (2022). Child executive function and future externalizing and internalizing problems: A meta-analysis of prospective longitudinal studies. Clin. Psychol. Rev..

[17] Campbell S.B., Shaw D.S., Gilliom M. (2000). Early externalizing behavior problems: Toddlers and preschoolers at risk for later maladjustment. Dev. Psychopathol..

[18] Hammerton G., Murray J., Maughan B., Barros F.C., Gonçalves H., Menezes A.M.B., Wehrmeister F.C., Hickman M., Heron J. (2019). Childhood behavioural problems and adverse outcomes in early adulthood: A comparison of brazilian and british birth cohorts. J. Dev. Life-Course Criminol..

[19] Racz S.J., King K.M., Wu J., Witkiewitz K., McMahon R.J., The Conduct Problems Prevention Research Group (2013). The predictive utility of a brief kindergarten screening measure of child behavior problems. J. Consult. Clin. Psychol..

[20] Bridgett D.J., Burt N.M., Edwards E.S., Deater-Deckard K. (2015). Intergenerational transmission of self-regulation: A multidisciplinary review and integrative conceptual framework. Psychol. Bull..

[21] Doebel S. (2020). Rethinking executive function and its development. Perspect. Psychol. Sci..

[22] Blair C., Raver C.C. (2015). School readiness and self-regulation: A developmental psychobiological approach. Annu. Rev. Psychol..

[23] Volckaert A.M.S., Noël M.P. (2015). Training executive function in preschoolers reduce externalizing behaviors. Trends Neurosci. Educ..

[24] Caporaso J.S., Boseovski J.J., Marcovitch S. (2019). The individual contributions of three executive function components to preschool social competence. Infant Child Dev..

[25] Rhoades B.L., Greenberg M.T., Domitrovich C.E. (2009). The contribution of inhibitory control to preschoolers’ social-emotional competence. J. Appl. Dev. Psychol..

[26] Garon N., Bryson S.E., Smith I.M. (2008). Executive function in preschoolers: A review using an integrative framework. Psychol. Bull..

[27] Binder A.S., Brown H.R., Harvey E.A. (2020). Executive Function and Trajectories of Emotion Dysregulation in Children with Parent-Reported Behavior Problems. J. Abnorm. Child Psychol..

[28] Maddio S., Greco C. (2010). Flexibilidad cognitiva para resolver problemas entre pares. ¿Difiere esta capacidad en escolares de contextos urbanos y urbanomarginales?. Rev. Interam. Psicol./Interam. J. Psychol..

[29] Holmes C.J., Kim-Spoon J., Deater-Deckard K. (2016). Linking executive function and peer problems from early childhood through middle adolescence. J. Abnorm. Child Psychol..

[30] Hughes C., Ensor R. (2008). Does executive function matter for preschoolers’ problem behaviors?. J. Abnorm. Child Psychol..

[31] Thorell L.B., Lindqvist S., Bergman Nutley S., Bohlin G., Klingberg T. (2009). Training and transfer effects of executive functions in preschool children. Dev. Sci..

[32] Diamond A., Lee K. (2011). Interventions shown to aid executive function development in children 4 to 12 years old. Science.

[33] Romero-López M., Pichardo M.C., Justicia-Arráez A., Cano-García F. (2021). Effect of the EFE-P program on the improvement of executive functions in Early Childhood Education. Rev. Psicodidactica..

[34] Martínez-Martínez A.M., Aguilar-Mejía O.M., Martínez-Villar S., Marino-García D. (2014). Caracterización y efectividad de programas de rehabilitación neuropsicológica de las funciones ejecutivas en pacientes con daño cerebral adquirido: Una revisión. Univ. Psychol..

[35] Kassai R., Futo J., Demetrovics Z., Takacs Z.K. (2019). A meta-analysis of the experimental evidence on the near- and far-transfer effects among children’s executive function skills. Psychol. Bull..

[36] Melby-Lervag M., Hulme C. (2013). Is working memory training effective? A meta-analytic review. Dev. Psychol..

[37] Melby-Lervåg M., Redick T.S., Hulme C. (2016). Working memory training does not improve performance on measures of intelligence or other measures of “far transfer” evidence from a meta-analytic review. Perspect. Psychol. Sci..

[38] Sala G., Gobet F. (2017). Working memory training in typically developing children: A meta-analysis of the available evidence. Dev. Psychol..

[39] Diamond A., Ling D.S. (2016). Conclusions about interventions, programs, and approaches for improving executive functions that appear justified and those that, despite much hype, do not. Dev. Cogn. Neurosci..

[40] Romero-López M., Pichardo M.C., Bembibre-Serrano J., García-Berbén T. (2020). Promoting Social Competence in Preschool with an Executive Functions Program Conducted by Teachers. Sustainability.

[41] Reynolds C.R., Kamphaus R.W. (1992). BASC: Behavior Assessment System for Children: Manual.

[42] González J., Fernández S., Pérez E., Santamaría P. (2004). Adaptación Española del Sistema de Evaluación de la Conducta en Niños y Adolescentes: BASC.

[43] Stroup W.W. (2012). Generalized Linear Mixed Models: Modern Concepts, Methods and Applications.

[44] Cohen J. (1988). Statistical Power Analysis for the Behavioral Sciences.

[45] Alba G., Fernández-Cabezas M., Justicia F., Pichardo M.C. (2015). The longitudinal effect of the Aprender a Convivir (learning to live together) programme in childhood: The development of social competence. Cult. Educ..

[46] Raaijmakers M.A., Smidts D.P., Sergeant J.A., Maassen G.H., Posthumus J.A., Van Engeland H., Matthys W. (2008). Executive functions in preschool children with aggressive behavior: Impairments in inhibitory control. J. Abnorm. Child Psychol..

